# Remission of relapsed/refractory classical Hodgkin lymphoma induced by brentuximab vedotin and pembrolizumab combination after allogeneic hematopoietic stem cell transplantation: a case report

**DOI:** 10.3389/fimmu.2024.1360275

**Published:** 2024-03-06

**Authors:** Federica Giannotti, Carmen De Ramon Ortiz, Federico Simonetta, Sarah Morin, Chiara Bernardi, Stavroula Masouridi-Levrat, Yves Chalandon, Anne-Claire Mamez

**Affiliations:** ^1^ Division of Hematology, Department of Oncology, Geneva University Hospitals and Faculty of Medicine, University of Geneva, Geneva, Switzerland; ^2^ Translational Research Center for Oncohematology, Department of Medicine, Faculty of Medicine, University of Geneva, Geneva, Switzerland

**Keywords:** Hodgkin lymphoma, pembrolizumab, brentuximab-vedotin, haploidentical allogeneic stem cell transplant, DLI

## Abstract

Allogeneic hematopoietic stem cell transplantation (allo-HSCT) is a potentially curative treatment option for patients with highly chemorefractory Hodgkin lymphoma (HL). The CD30-targeting antibody-drug conjugate Brentuximab-Vedotin (BV) and programmed cell death protein-1 (PD-1) blocking agents have demonstrated clinical activity with durable responses in relapsed/refractory (r/r) HL. However, patients with a history of allo-HSCT were frequently excluded from clinical trials due to concerns about the risk of graft-versus-host disease (GVHD). We report the clinical history of a patient with refractory classical HL who underwent two allo-HSCTs (first from matched unrelated and second from haploidentical donor) after relapsing on BV and nivolumab and for whom durable remission was finally obtained using BV-pembrolizumab combination for relapse after haploidentical HSCT. Such treatment was associated with the onset of GVHD after only two cycles which led to treatment discontinuation. However, the side effects were rapidly controlled, and after 2 years of follow-up, the patient is still in remission. Our data support the feasibility and efficacy of combining PD-1 blockade with BV to enhance the graft-versus-lymphoma effect after allo-HSCT.

## Introduction

Treatment options for hodgkin lymphoma (HL) relapsing after autologous transplant include BV and checkpoint inhibitors (CPIs) (1–3). Despite these advances, patients hardly achieve long-term disease control without the use of allogeneic hematopoietic stem cell transplantation (allo-HSCT). This procedure has now been used in relapsed/refractory (r/r) HL for more than 30 years and the advent of reduced-intensity conditioning (RIC) has led to substantial improvement in non-relapse mortality (NRM) (4–6). Nevertheless, relapse after allo-HSCT often occurs due to immune evasion and loss of the graft-versus-lymphoma (GvL) effect (7). Restoring the GvL effect is an appealing strategy to treat relapse after allo-HSCT, which has otherwise dismal outcomes. Donor lymphocyte infusion (DLI) has been shown to induce high response rates and durable salvage in post-transplant relapse (8). Preclinical studies have demonstrated the effectiveness of CPIs in increasing the GvL effect (9). Still, there is the concern that checkpoint blockade might dramatically increase the risk of graft-versus-host disease (GvHD) (10). Brentuximab-vedotin (BV) in association with anti-PD-1 monoclonal antibodies has been shown to be effective in the treatment of r/r HL, mostly in the pre-transplant setting (11). We report the case of a r/r HL relapsing after two allo-HSCTs who rapidly responded to BV-pembrolizumab combination therapy without developing major complications.

## Case report

**Figure 1:**
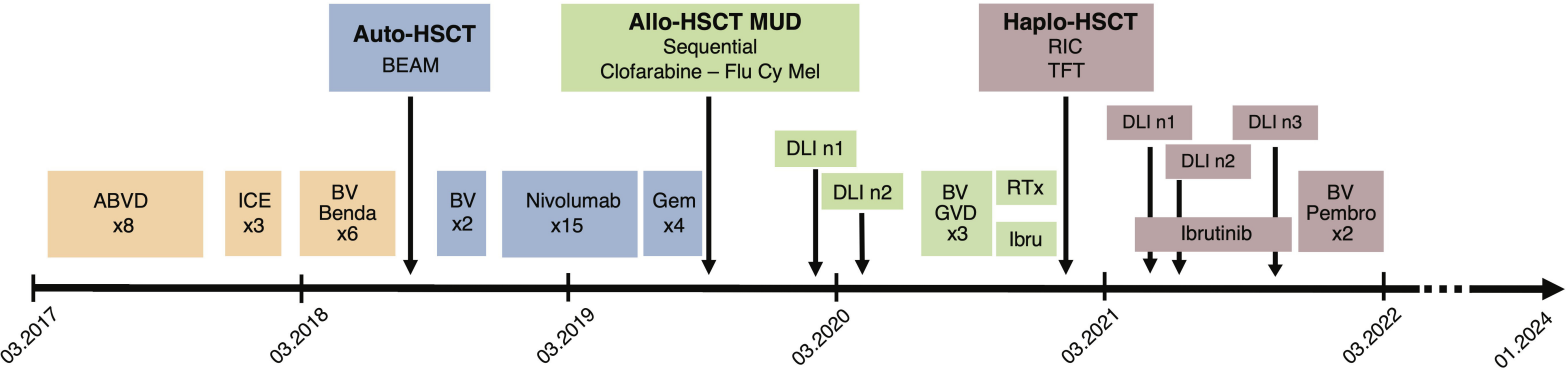
Summary of the antitumor treatments received by the patient since diagnosis in March 2017. ABVD, Adriamycin, bleomycin, vinblastine, dacarbazine; ICE, ifosfamide, carboplatin, etoposide; BV, Brentuximab-Vedotin; Benda, bendamustine; Auto-HSCT, autologous hematopoietic stem cell transplantation; BEAM, carmustine, etoposide, cytarabine, melphalan; Gem, Gemcitabine; Allo-HSCT, allogeneic hematopoietic stem cell transplantation; MUD, matched unrelated donor; Flu Cy Mel, fludarabine, cyclophosphamide, melphalan; DLI, Donor lymphocyte infusion; GVD, gemcitabine, vinorelbine, liposomal doxorubicine; RTx, radiotherapy; Ibru, ibrutinib; Haplo-HSCT, haploidentical hematopoietic stem cell transplantation, RIC, reduced intensity conditioning; TFT, thiotepa fludarabine treosulfan; Pembro, pembrolizumab.

**Figure 2:**
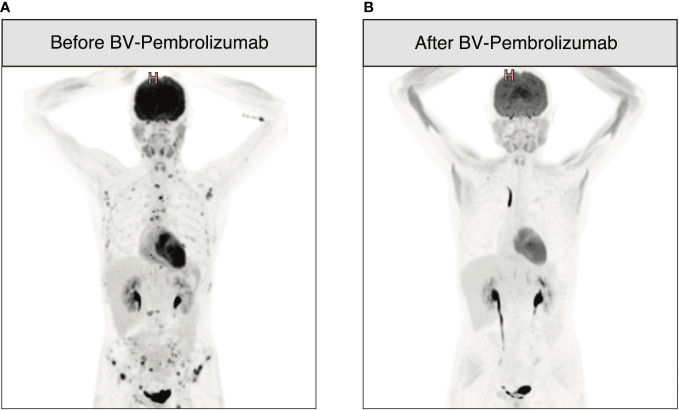
18F-fluorodeoxy-glucose–positron-emission tomography (FDG-PET)-CT images before and after therapy with BV-pembrolizumab. **(A)** Progression of nodal and bone lesions 11 months after haplo-HSCT and three doses of DLI; **(B)** PET-CT performed after two cycles of BV 1.8 mg/kg in combination with pembrolizumab 100 mg every 3 weeks showing CR of HL as per **Figure 1**.

An 18-year-old man was diagnosed with classical HL, nodular sclerosis subtype (NS), initial stage IIIB (according to Lugano classification), and an IPS score of 2/7 in March 2017. He presented with bulky disease with an anterior mediastinal mass (10x6 cm), multiple lymph nodes above the diaphragm, and spleen involvement. The patient initially responded to two cycles of ABVD (adriamycin, bleomycin, vinblastine, and dacarbazine) chemotherapy, and after eight cycles, presented a relapse confirmed by biopsy, with supradiaphragmatic lymph node involvement. Three cycles of ICE (ifosfamide, carboplatin and etoposide) salvage chemotherapy led to a partial response (PR). After autologous stem cell collection, the patient received a third line with BV-bendamustine with a positron emission tomography/computed tomography (PET/CT) after six cycles showing a refractory disease with a Deauville score of four. An autologous stem cell transplant was performed in July 2018 after high-dose BEAM (carmustine, etoposide, cytarabine, and melphalan) conditioning (**Figure 1** summarizes the antitumor treatments received). PET/CT at 1 month after autologous HSCT showed disease progression. Two additional cycles of BV monotherapy were administrated with subsequent progression and new celiac and retroperitoneal node involvement. BV was stopped and 15 cycles of nivolumab monotherapy every 2 weeks were administered. The patient had a significant clinical improvement, and a CT scan initially showed a reduction in the lymph nodes. However, PET/CT evaluation after 15 cycles revealed further disease progression with a Deauville score of five (supra and subdiaphragmatic nodes and multinodular spleen involvement). The patient was then addressed to our center to undergo allo-HSCT. A bridging with gemcitabine 1000 mg/m^2^ per week for 1 month led to a partial response (Deauville score of four). A sequential conditioning regimen (clofarabine, cyclophosphamide, fludarabine, and melphalan) was administered and peripheral blood stem cells from a matched unrelated donor (MUD) were infused in August 2019 (**Figure 1**). GvHD prophylaxis consisted of anti-thymoglobulin (ATG), cyclosporine A (CsA), and mycophenolate mofetil (MMF). The patient did not develop any major transplant-related complications. Immunosuppression was stopped 3.5 months after HSCT. Full donor chimerism was measured in peripheral blood starting from day 30 post-transplant. PET/CT at 2 and 4 months after allo-HSCT showed complete remission (CR). A first prophylactic donor lymphocyte infusion (DLI) was performed at 5 months post-HSCT, at a CD3 dose of 10^6^/kg. PET/CT at 7 months post-HSCT showed suspicious hypermetabolism of the Waldeyer ring, bilateral cervical nodes, an iliac node, and two lung nodules, with negative infectious screening and negative cervical node biopsy for HL. A second DLI dose (CD3 5x10^6^/kg) was given at 8 months post-HSCT. Chest low-dose CT scan was performed once a month showing a significative morphological progression of the lung nodules despite empirical antibiotic therapy. Bronchioalveolar lavage and endobronchial ultrasound-guided transbronchial needle aspiration were performed twice, showing no tumor cells and revealing parainfluenzae virus first, then *Actinomyces oris* and *graevenitzii*. At 9 months post-transplant, PET/CT showed hypermetabolic sub- and supradiaphragmatic lymph nodes and a clear morpho-metabolic progression of the lung masses. Diagnostic upper right lobectomy confirmed HL relapse with CD30-positive Reed-Sternberg cells. Salvage chemotherapy with GVD (gemcitabine, vinorelbine, and liposomal doxorubicine) and BV was administrated for three cycles, during which the patient developed toxic cryptogenic organizing pneumonia (COP) treated with steroids. PET/CT after three cycles showed a dissociated response with reduced hypermetabolism of supradiaphragmatic nodes and further morpho-metabolic progression of the subdiaphragmatic ones. Indication of a second HSCT from a haplo-identical donor was discussed and the patient was treated with radiotherapy on subdiaphragmatic sites administered as 20 fractions of Volumetric-Modulated Arc Therapy, for a total of 36Gy, in combination with ibrutinib 140 mg/day (12, 13). PET/CT 1 month after the end of radiotherapy and 2 months after starting ibrutinib showed a PR, with morpho-metabolic regression of the irradiated nodes. The patient underwent haploidentical HSCT with peripheral blood stem cells from his father in December 2020, after a reduced intensity conditioning with thiotepa, fludarabine, and treosulfan (**Figure 1**). GvHD prophylaxis consisted of post-transplant cyclophosphamide (PTCy), MMF till day 35, and tacrolimus withdrawn 3 months post-transplant. Full donor chimerism was obtained. PET/CT at 2 months after the second transplant showed CR. Maintenance therapy with ibrutinib 280 mg/day was started at 3.5 months after the second allo-HSCT. Two weeks later, two hepatic hilar hypermetabolic nodes appeared, and 1 month later, a PET/CT showed disease progression with right mediastinal pleura hypermetabolism (9.6x3.8mm), a mammary node, increased involvement of hepatic nodes, and an iliac bone lesion. DLI at a CD3 dose of 10^6^/kg was given and PET/CT performed 1.5 months after showed a dissociated response with progression of multiple bone lesions of the axial skeleton and morpho-metabolic regression of supradiaphragmatic lymph nodes. A CT-guided biopsy of the iliac bone did not reveal HL infiltration, reporting only a likely reactional CD8+ T cell population. A second dose of DLI (5x10^6^/kg) was administered at 7 months post-transplant, and a PET/CT 1 month later showed morphological stability but with metabolic progression of several supradiaphragmatic nodes (cervical, axillary, and clavicular) and regression of some of the bone lesions while others persisted. A PET/CT 3 months later showed frank disease progression. A third dose of DLI was given (10^7^/kg) and ibrutinib continued for 1 additional month and then stopped due to documentation of further nodal and bone progression on PET/CT (**Figure 2A**). Bone marrow biopsy showed infiltration with classical HL nodular sclerosis subtype. The patient did not present cytopenia or mixed chimerism despite the diffuse bone involvement. At this time, a clinical diagnosis of COP relapse required the reintroduction of prednisone 20 mg/day. Salvage treatment with BV 1.8 mg/kg in combination with pembrolizumab, at a reduced dose of 100 mg every 3 weeks trying to reduce toxicity (14–16), was started at the beginning of December 2021. At day 22 of the second cycle, moderate overlap GvHD (skin, genital, eyes, and mouth) was documented, and this was despite the ongoing prednisone treatment for the COP. A PET/CT scheduled the same day showed morpho-metabolic regression of the nodal and bone lesions revealing a CR (Deauville score of two) of HL (**Figure 2B**). No additional significant toxicities related to the treatment were observed. For GvHD treatment, oral prednisone was increased (0.5 mg/kg/day) and topical treatment was introduced, leading to only a partial response. Ruxolitinib was then introduced in June 2022, which induced complete remission of GvHD and allowed prednisone tapering with complete withdrawal in September 2022. Ruxolitinib was stopped in April 2023. Two years since the introduction of BV-pembrolizumab, the patient has remained in CR of HL and does not present any signs of GvHD.

## Discussion and conclusions

In this case report, we highlighted the potential of the administration of pembrolizumab in combination with the anti-CD30 antibody-drug conjugate BV post-allo-HSCT in r/r HL. Our patient achieved durable remission after only two cycles of therapy with a low dose of pembrolizumab. The onset of GvHD led, however, to treatment interruption and was rapidly controlled by prednisone and ruxolitinib.

Immune exhaustion, a state of reduced effector function and proliferation of T cells overexpressing given markers such as programmed cell death protein 1 (PD-1) and associated with chronic antigen exposure (17), has been associated with GvL effect impairment after allo-HSCT (18). Immunosuppression by immune-checkpoint ligand expression at the tumor surface combined with upregulation of checkpoint molecules, including PD-1, at the T cell surface during immune reconstitution after allo-HSCT (19, 20) is one of the mechanisms of immune escape, potentially leading to post-transplant disease relapse (7).

DLI is an established therapeutic option to restore the GvL effect for hematological malignancies relapsing after HSCT, including HL (21–23). It is believed that DLI acts by normalizing the T cell receptor repertoire and clonal expansion of allogeneic T cells and improving coordination of T and B cell immunity. Bachireddy et al. showed that response to DLI is associated with a preexisting reservoir of antitumor CD8+ T cells residing at the tumor site, to which CD4+ DLI provides immunologic help, not only to expand this reservoir but also to reverse T-cell exhaustion (24). The presence of T-cell exhaustion may indicate that this reservoir exists. In contrast, in the absence of such a reservoir, a lack of DLI response is associated with both insufficient quantities of infiltrating T cells and the absence of phenotypic evidence of past T-cell activation. They also suggest the use of immune-activating therapies that may reverse T-cell exhaustion, such as CPIs, having the potential to restore the GvL effect even when DLI fails (24). Other studies have shown that loss of GvL effect is associated with increased PD-1 expression and reduced oligoclonal expansion in allogeneic transplant recipients and can be effectively restored by PD-L1 blockade (25). This suggests that PD-1 blockade may act through distinct pathways of immunomodulation, which is potentially more effective in reactivating the GvL effect. Blocking the PD1/PD-L1 interaction in malignancies that acquire PD-L1 as an adaptative response to immune pressure relieves T cell inhibition exerted by tumor cells on CD8+ T cells against minor histocompatibility antigens (26). Early-phase clinical trials have shown the efficacy and feasibility of PD-1 blockade in the post-allo-HSCT relapse setting (27, 28). Our data support the potential of CPIs even after DLI failure and suggest a potential synergistic effect between allo-HSCT and checkpoint blockade therapy. In fact, our patient had already received CPIs before HSCT without any effect, probably due to other mechanisms of immune tolerance between host T cells and HL cells.

Unfortunately, anti-PD-1 therapies can increase the incidence and severity of GvHD and other immune complications, mainly due to a loss of donor-CTL regulation mediated by PD-L1 expressed on host antigen-presenting cells (9). Their high potential toxicities should therefore not be underestimated. It has been reported that PD-1 blockade in relapsed HL allo-HSCT patients can be frequently complicated by the rapid onset of severe and treatment-refractory GvHD and that the use of PD-1 blockade in the allo-HSCT setting may be associated with increased toxicity and/or increased efficacy, and the balance of those two effects is likely to significantly affect patient outcomes (29–32). This underlines the need to develop effective prevention strategies and new approaches to separate the GvL effect and GvHD. For example, the use of PTCy as GvHD prophylaxis has been shown to reduce the risk of complications in patients treated with CPIs before HSCT (33, 34). Our patient did not develop post-DLI GvHD despite the high doses of CD3 received after both HSCTs, while GvHD appeared after only two cycles of BV-pembrolizumab, leading to therapy withdrawal. Importantly, the use of reduced doses of pembrolizumab to minimize the risk of GvHD did not prevent the development of this immune complication. Nevertheless, GvHD was easily controlled by low doses of prednisone in association with ruxolitinib. In addition, ruxolitinib has been shown to have some, although limited, activity against HL cells (35). We can hypothesize that ruxolitinib has played a role in maintaining our patient in CR of HL when used as an anti-GvHD second-line treatment after stopping the BV-pembrolizumab administration. At the time of this report, ruxolitinib has been stopped for 8 months without evidence of disease recurrence.

Although CPIs were the cornerstone of success in our patient, we believe that donor choice also played a role in the immune response observed. A retrospective analysis by the EBMT on 2204 patients with r/r HL undergoing allo-HSCT suggested improved outcomes with more contemporary transplant practices with an increase in the use of haploidentical donors, reduced intensity conditioning (RIC), and peripheral blood as stem cell source (36). In fact, haplo HSCT has shown very promising results in patients with HL including r/r cases compared to other types of donors, probably due to a stronger GvL effect exerted in this peculiar immunological setting (37–41). It is our belief that this “haplo” effect could be empowered by PD-1 blockade by CPIs leading to an enhanced graft-versus-Hodgkin effect. This explains our choice to go for a second HSCT from a haplo donor before using CPIs rather than employing CPIs after the first MUD allo-HSCT.

Both pembrolizumab and BV have shown encouraging results in patients with HL relapsing after autologous transplantation and are approved in this setting (1, 2). Direct comparison of pembrolizumab with brentuximab showed an advantage of the former in progression-free survival (PFS) (42). Several studies showed brentuximab with DLI for HL relapsing after allo-HSCT to be effective with minimal toxicity, hypothesizing the immunomodulating role of BV in preventing GvHD (43, 44). A recent study highlights the use of pembrolizumab to induce the GVL effect in relapsed hematologic malignancies after HSCT (28). The association of both agents has rarely been described in the literature but has mostly been described in r/r HL as a bridge to autologous transplant (11). Our goal in combining pembrolizumab and BV was to unleash the activation of donor lymphocytes using CPIs while favoring epitope spreading through tumor lysis using a targeted therapy to maximize the anti-tumor effect.

In conclusion, this case supports the efficacy of PD-1-blockade associated with targeted therapy in successfully overcoming T-cell exhaustion after allo-HSCT, resulting in long-term remission of a highly refractory disease without significant toxicity. Our data suggest the haploidentical setting may have favored these results and that lower doses of CPIs should be considered to reduce immune complications.

## Data availability statement

The original contributions presented in the study are included in the article/Supplementary Material. Further inquiries can be directed to the corresponding author.

## Ethics statement

Written informed consent was obtained from the individual(s) for the publication of any potentially identifiable images or data included in this article.

## Author contributions

FG: Writing – original draft. CD: Writing – review & editing. FS: Writing – review & editing. SM: Writing – review & editing. CB: Writing – review & editing. SM: Writing – review & editing. YC: Writing – review & editing. AM: Writing – review & editing.
